# From Thesis to Publication: A Five-Year Cross-Disciplinary Analysis in Pathology, Urology, and Endocrinology (2018-2022)

**DOI:** 10.5146/tjpath.2026.14783

**Published:** 2026-01-31

**Authors:** Esra Betul Tunce, Busra Yaprak Bayrak, Mahmut Akgul

**Affiliations:** Department of Pathology, Kocaeli University School of Medicine, Kocaeli, Türkiye; Atlas University School of Medicine, Istanbul, Türkiye; Brigham and Women’s Hospital, Boston, MA, USA

**Keywords:** Medical residency, Thesis publication, Scientific output

## Abstract

**Objective:** Despite the legal requirement to complete a thesis during residency training in Türkiye, the extent to which these theses are translated into high-quality scientific publications remains unclear. Disciplinary differences in research culture, resource availability, and clinical workload may influence these outcomes.

**Material and Methods:** This cross-sectional study analyzed 1245 open access residency theses completed between 2018 and 2022 in the fields of pathology (n=344), endocrinology (n=525), and urology (n=376). Theses were retrieved from the National Thesis Center of the Council of Higher Education. Their publication status was identified via searches in PubMed and Google Scholar. Data collected included journal index status (SCI-E, ESCI, ULAKBIM), Journal Impact Factor™ (JIF), citation count, and time to publication. Statistical comparisons were made using chi-squared and Kruskal-Wallis tests with p < 0.05 considered significant.

**Results:** Among the 1245 residency theses analyzed, 344 (27.6%) were in pathology, 525 (42.2%) in endocrinology and metabolic diseases, and 376 (30.2%) in urology. The conversion rate to publication significantly differed across specialties (p = 0.0002): 86 of 344 pathology theses (25.0%), 115 of 525 endocrinology theses (21.9%), and 139 of 376 urology theses (37.0%) were published. Urology theses had the highest representation in SCI-E indexed journals (72.7%), while endocrinology demonstrated the highest mean Journal Impact Factor (2.3; p < 0.0001). The average number of citations per publication was also highest in urology (4.5), although this difference was not statistically significant (p = 0.0673). Median time to publication ranged from 2.3 to 2.7 years, with no significant difference between specialties (p = 0.1287). Differences in the distribution of Q2, Q3, and Q4 journal publications were statistically significant between specialties.

**Conclusion:** Endocrinology had the highest number of theses, whereas urology had the highest publication rate and number of citations per publication.

## INTRODUCTION

Medical education in Türkiye is a six-year program and includes a final internship year (1). Graduates who successfully complete this process are eligible to apply for residency training with the Medical Specialization Training Entrance Exam (in Turkish, *Tipta Uzmanlik Sinavi*, *TUS*) (2). Specialization training is carried out in university hospitals or training and research hospitals affiliated with the Turkish Ministry of Health and can last 3 to 5 years depending on the specialty (e.g. 4 years for pathology, 5 years for urology, or 3 years for endocrinology after completion of internal medicine residency) (3,4).

One of the required components of the post-graduation training in Türkiye is a successful completion of a clinical research project and to successfully defend and finalize its thesis. Residents are required to initiate a clinical research project in the first half of their training and to conduct their research under the supervision of a faculty committee (3). Previous national and international studies consistently show that only a limited proportion of residency theses are converted into academic publications (4). For example, in a multicenter study analyzing the theses and publication data of 231 physicians who completed pathology residency training in France, it was reported that 48% of the theses turned into scientific publications; however, only 18.2% were published in PubMed indexed journals (5). In a study conducted in the field of infectious diseases and clinical microbiology in Turkey, it was determined that only 17.8% of 870 theses prepared between 2000 and 2020 were published in internationally indexed journals (6). Similarly, in another study conducted in the field of periodontics, only 47% of residents published their theses in any publication and only 29.48% of these publications were published in journals covered by SCI-Expanded (7).

The conversion of residency theses into publications influences both individual academic development and national scientific productivity, and this rate varies substantially across medical disciplines (e.g. pathology, urology, endocrinology) (1,5). These differences may be related to many variables such as clinical workload, research culture, the level of supervisor support, and individual pursuit of academic or non-academic career (6).

The aim of this study was to examine the publication rates of residency theses defended between 2018 and 2022 in pathology, urology, and endocrinology in Türkiye, using an interdisciplinary comparative approach. In selecting pathology, urology, and endocrinology for comparison, we intentionally included three specialties that represent distinct domains of medical practice in Türkiye: a diagnostic field (pathology), a surgical field (urology), and an clinical field (endocrinology). These specialties differ substantially in research culture, workload dynamics, and academic output patterns, making them suitable for an interdisciplinary evaluation of thesis-to-publication performance.

## MATERIALS AND METHODS

### Study Design

This study is a cross-sectional, descriptive analysis that aims to evaluate the conversion rate of theses prepared within the scope of residency training in Turkey into peer reviewed publications and the qualitative characteristics of the publications. The study covers three different medical specialties (pathology as diagnostic specialty, urology for surgical specialty, and endocrinology for clinical specialty) and covers the five-year period between 2018 and 2022.

**Figure 1:**
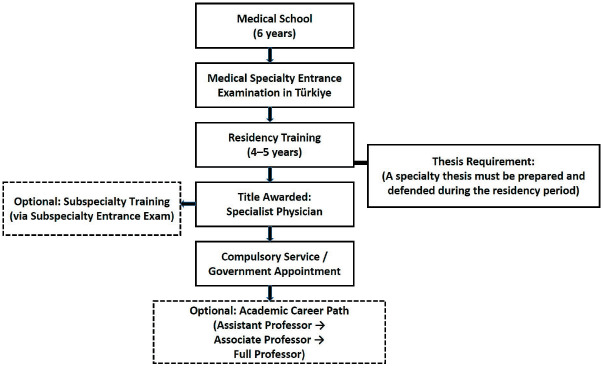
Overview of the medical and academic career paths in Türkiye . The flowchart illustrates the stages from medical school to academic promotion, including specialization training and subspecialty options.

To facilitate comprehension of the structure and stages of medical specialization training in Türkiye, a schematic representation is provided in Figure 1, outlining the general progression from undergraduate medical education to academic career advancement.

### Thesis Selection

The online database of the Council of Higher Education National Thesis Center (8) (https://tez.yok.gov.tr/UlusalTezMerkezi/) was used in the data collection process. All successfully completed residency theses are submitted to this website and are accessible to the public as an abstract and/or full text. In this digital, open-access database, all completed residency theses in “pathology”, “urology” and “endocrinology” between January 1, 2018 and December 31, 2022 were evaluated. Only theses that were open to access and whose full texts could be accessed were included in the study. Duplicate records, theses with incomplete data, and incorrectly classified records belonging to different specialties were excluded from the study.

### Determining Publication Status

For each selected thesis, PubMed (9) and Google Scholar (10) databases were searched to determine whether the relevant study was published as a peer-review article. During the search, both thesis title (in Turkish and English if available) and the names of the thesis author and thesis advisor(s) were searched. Theses that were converted into publications were defined as publications that were accepted as original research articles and that included the thesis author and advisor in the authorship.

### Data Collection

The following variables for each thesis were recorded: the field of expertise (branch) related to the thesis topic; whether the thesis was published as an article (Yes/No); the title of the published study and the journal name; whether the relevant journal was indexed within the Web of Science Core Collection; the Journal Impact Factor™ (JIF) value of the journal for 2023 and the impact factor quartile corresponding to this value (JIF Quartile, Q1-Q4); the PubMed ID (PMID) of the publication, if any; the publication date of the article (day/month/year); the total number of citations the publication received in the Google Scholar database; the period between the date the thesis was defended and the date the article was published (in months).

### Data Analysis

All statistical analyses were performed to evaluate associations, differences, and trends across categorical and continuous variables of the publication rates, distribution by branches, journal index status, and citation numbers. The Chi-squared test for independence was used to examine relationships between categorical variables, such as departmental differences in publication rates. In cases where the categorical variable had an inherent order, such as journal quartiles (Q1-Q4), a global Chi-square test was applied to compare quartile distributions across specialties. The Kruskal-Wallis test, a nonparametric alternative to ANOVA, was used to compare medians across three or more independent groups for non-normally distributed data, including variables such as citation counts, time between thesis completion and publication, and journal impact factors. When the Kruskal-Wallis test indicated significance, Dunn’s multiple comparisons test was conducted post-hoc to identify which specific group differences were statistically significant, with adjustments for multiple testing. Descriptive statistics, including frequencies and percentages for categorical variables and means with minimum and maximum values for continuous data, were also presented. All statistical analyses were conducted using the GraphPad Instat Statistical Program, and a p-value less than 0.05 was considered statistically significant.

## RESULTS

A total of 1245 residency theses from pathology, endocrinology, and urology specialties between 2018 and 2022 were included in the study. Of these, 344 (27.6%) were in pathology, 525 (42.2%) in endocrinology and metabolic diseases, and 376 (30.2%) in urology. Among the analyzed theses, 25.0% of pathology theses (n=86), 21.9% of endocrinology theses (n=115), and 37.0% of urology theses (n=139) were subsequently published as scientific articles. The difference in publication rates among specialties was statistically significant (p = 0.0002). The majority of these were published in SCI-E-indexed journals (pathology: 73.3%, endocrinology: 48.7%, urology: 72.7%), followed by ESCI-indexed journals (7.0%, 13.0%, 12.9% respectively), ULAKBIM-indexed national journals (14.0%, 26.1%, 12.2%), and other non-indexed journals (5.8%, 12.2%, 2.2% respectively).

In terms of scientific impact, the average number of citations per article was lowest in pathology (2.2, range 0-24), compared to endocrinology (4.0, range 0-66) and urology (4.5, range 0-54), although this difference did not reach statistical significance (p = 0.0673). The average time between thesis completion and publication was also not significantly different across groups (pathology: 2.7 years, endocrinology: 2.4 years, urology: 2.3 years; p = 0.1287). The number of journals with a specified Journal Impact Factor™ (JIF) in which these theses were published was 69 for pathology, 66 for endocrinology, and 119 for urology. A statistically significant difference was observed in the average JIF values among specialties: 1.4 (range 0.1-4.8) in pathology, 2.3 (range 0.2-6.1) in endocrinology, and 2.0 (range 0.1-25.3) in urology (p < 0.0001). The groups showing statistically significant pairwise differences as revealed by the Post-hoc Dunn’s test are denoted with superscript letters in Table 1.

**Table 1:** General distribution of theses and related publications across specialties

**Parameter**	**Pathology** **(N=344)**	**Endocrinology and Metabolic Diseases (N=525)**	**Urology** **(N=376)**	**p value**
Number of Published Theses (n, %)	86 (25.0)	115 (21.9)	139 (37.0)	**0.0002**
Distribution of Publication Types (n, %) Number of Publications in SCI-E-Indexed Journals (Web of Science Core Collection)	63 (73.3)	56 (48.7)	101 (72.7)	
Number of Publications in ESCI-Indexed Journals (Web of Science Core Collection)	6 (7.0)	15 (13.0)	18 (12.9)	
Number of Publications in National Journals Indexed in ULAKBIM	12 (14.0)	30 (26.1)	17 (12.2)	
Number of Publications in Other Journals	5 (5.8)	14 (12.2)	3 (2.2)	
Average Number of Citations per Publication (min-max)	2.2 (0-24)	4.0 (0-66)	4.5 (0-54)	0.0673
Average Time Between Thesis Completion and Publication (years, min-max)	2.7 (0.3-7)	2.4 (0.5-7)	2.3 (0.1-6)	0.1287
Number of Journals with a Specified Web of Science Journal Impact Factor™ (JIF)	69	66	119	**<0.0001**
Average Web of Science Journal Impact Factor™ (JIF) (min-max)	1.4 (0.1-4.8)a	2.3 (0.2-6.1)b	2.0 (0.1-25.3)b	

a,b: Superscript letters indicate statistically significant pairwise differences based on Dunn’s post-hoc test. Groups that do not share the same letter differ significantly at p < 0.05.

Table 2 presents the quartile distributions of SCI-E-indexed journal publications. The most frequent quartile in pathology was Q4 (39.7%), while endocrinology publications were predominantly in Q2 (33.9%) and Q3 (32.1%). Urology publications were mostly in Q3 (52.5%). A global Chi-square test comparing quartile distributions across specialties showed a statistically significant overall difference (p = 0.018).

**Table 2:** Quartile (Q) ranking of SCI-E indexed journal publications by specialties

**Quartile (Q)**	**Pathology (N=63)**	**Endocrinology and Metabolic Diseases (N=56)**	**Urology (N=101)**	Overall p value*
Q1 (n, %)	4 (6.3)	8 (14.3)	11 (10.9)	**0.018**
Q2 (n, %)	11 (17.5)	19 (33.9)	35 (34.7)	
Q3 (n, %)	23 (36.5)	18 (32.1)	53 (52.5)	
Q4 (n, %)	25 (39.7)	11 (19.6)	20 (19.8)	

* Overall p-value was calculated using a global Chi-square test comparing quartile distributions across specialties

## DISCUSSION

This study aimed to evaluate the rate and characteristics of scientific publications derived from residency theses in three distinct medical specialties in Türkiye. In pathology, 25% of the 344 theses evaluated were converted into publications. Among these, 73.3% were published in SCI-E-indexed journals, with a relatively even distribution across quartiles, though Q3 and Q4 journals were predominant. The average citation count per publication was 2.2, and the mean time to publication following thesis defense was 2.7 years. In contrast to our findings, Belgaid and Moktefi reported a 48% publication rate among pathology theses in France (5). However, their assessment of visibility was based solely on PubMed indexing (18.2%), and citation data were not provided. Therefore, direct comparison of research visibility and impact between the two countries is limited by the use of different metrics.

In endocrinology, although the publication rate (21.9%) was slightly lower than for pathology, the average citation count (4.0) and journal impact factors (mean JIF 2.3) were notably higher. This suggests a tendency for endocrinology theses to result in higher-quality publications. Endocrinology theses also demonstrated a slightly shorter mean conversion period (2.4 years). These outcomes parallel those reported in Kapsetaki, where neuroscientific research with translational or clinical implications often achieved faster and higher-impact publication rates (11). It should also be emphasized that endocrinology is a subspecialty within internal medicine, and its residency training structure, clinical workload, and academic environment differ from those of pathology and urology. These structural differences may influence research productivity and publication behaviors and may partly explain the higher citation impact observed in endocrinology-derived publications.

Urology stood out with the highest publication rate (36.9%) and average number of citations per publication (4.5), albeit with a comparable mean time to publication (2.3 years). Notably, a significant proportion of SCI-E publications in urology appeared in Q2 and Q3 journals, echoing trends reported by Altenberger et al., who analyzed German surgical residency theses and highlighted urology as a field with robust translational research potential and established publication pipelines (12). Quartile distributions were evaluated using a single global Chi-square test to avoid multiple testing and Type I error inflation, providing a more reliable comparison across specialties.

Across all fields, a considerable number of articles (17-49 depending on the specialty) were published in journals without a listed impact factor in the Web of Science, raising concerns about the transparency and academic rigor of certain dissemination pathways. Moreover, despite the legal and academic requirement to produce a thesis during residency training in Türkiye (1,3), the relatively low publication rates (especially in pathology and endocrinology) underscore the gap between mandatory research efforts and their scientific translation (5–7).

Comparing our results with national data, İrkören et al. reported a 17.8% publication rate of infectious disease theses in Türkiye between 2000 and 2020 (6). Karayürek et al. found similar trends in periodontology, where only 29.5% of publications reached SCI-E-indexed journals (7). The present study, covering a more recent period and multiple fields, demonstrates that while progress has been made—especially in urology—systemic challenges persist.

Several factors may underlie the variability in publication success, including institutional research culture, availability of mentoring, publication pressure, and specialty-specific feasibility of clinical research (7,13). Moreover, differences in research design—such as clinical versus experimental or morphological studies—could influence citation trajectories and publication feasibility; however, methodological subclassification was not possible due to inconsistent reporting in the publicly available thesis files. The higher impact and citation rates observed in endocrinology and urology may reflect stronger infrastructure or international collaboration networks (14), while the lower figures in pathology may be partially due to limited access to multicentric datasets or advanced methodologies like molecular pathology (5). These findings are consistent with our own results, in which pathology theses had lower mean citation counts and impact factor averages compared to other specialties.

Our findings also highlight a concerning lag time between thesis defense and publication, averaging over two years across specialties. While this delay is not unique to Türkiye —as reflected in data from France, Slovakia, and Romania (5,14–16)—it reinforces the need to better integrate publication planning into the thesis process. In addition, newly graduated specialists in Türkiye are obligated to complete a mandatory civil service period immediately after residency, often under heavy clinical workload and in newly assigned institutions. This can significantly limit the time and resources available for manuscript preparation, revision, and submission, thereby contributing to the prolonged thesis-to-publication interval observed across all specialties. Structured research mentorship, early journal targeting, and writing support programs could enhance output efficiency.

This study has several limitations. First, the identification of thesis-derived publications was limited to three databases—PubMed, Google, and Google Scholar—which may have resulted in the omission of articles indexed exclusively in other platforms such as Scopus or regional journals. We evaluated the indexing status of journals only within the Web of Science Core Collection (SCI-E, ESCI) and ULAKBIM databases, and the journal quartile rankings (JIF Quartile) were reported exclusively for SCI-E-indexed publications, as ESCI and national journals do not have established quartile metrics. Therefore, comparisons involving journal quality and impact should be interpreted with caution. Another limitation is that only three specialties—pathology, endocrinology, and urology—were included, which, although chosen for their differing academic dynamics, restricts the generalizability of the findings to other medical disciplines. Additionally, the process of identifying matching publications relied on manual searches based on thesis titles and author names, which may not capture publications with significantly altered titles or modified authorship order. In addition, international comparisons—such as those involving the French pathology cohort—should be interpreted cautiously, as citation metrics were not reported in these external datasets, preventing direct evaluation of cross-country differences in research visibility or academic impact. Lastly, citation counts and impact factors were recorded at a single time point, and thus do not reflect changes in academic influence over time. Despite these constraints, the study offers valuable insight into thesis-to-publication trends and highlights the need for structured academic support during specialty training in Türkiye .

This study offers a comprehensive, cross-disciplinary perspective on the scientific conversion of medical specialization theses in Türkiye. While urology stands out in terms of publication performance, all three fields reveal significant room for improvement in transforming mandated research into high-impact scientific output. Cultivating a robust, publication-oriented academic environment during specialty training could substantially enhance both national academic productivity and international research visibility.

## Ethical Approval

The study protocol was approved by the Ethics Committee of Non-invasive Clinical Research of Kocaeli University (GOKAEK-2025/18/08).

## Conflict of Interest

The authors declare that they have no competing interests.
